# Supplementary material for: Hepatic Expression of ACBP Is a Prognostic Marker for Weight Loss After Bariatric Surgery
Source: Biomolecules. 2025 Aug 16;15(8):1173. doi: 10.3390/biom15081173 (PMC12384758; doi:10.3390/biom15081173)
Supplement: Supplementary file 1 [file biomolecules-15-01173-s001.zip › biomolecules-3786304-supplementary.pdf]

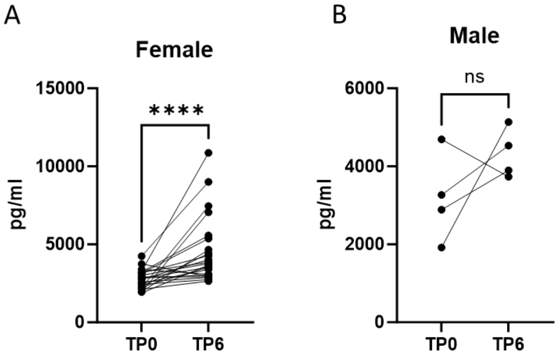

Supplementary Figure S1. (A, B) Serum levels of ACBP/DBI increase significantly 6 months after bariatric surgery in female (A), n=24 but not in male patients (B) n=4 Statistical significance was assessed using a Wilcoxon matched-pairs signed rank test after a Grubb's test for outlier detection (A, B).

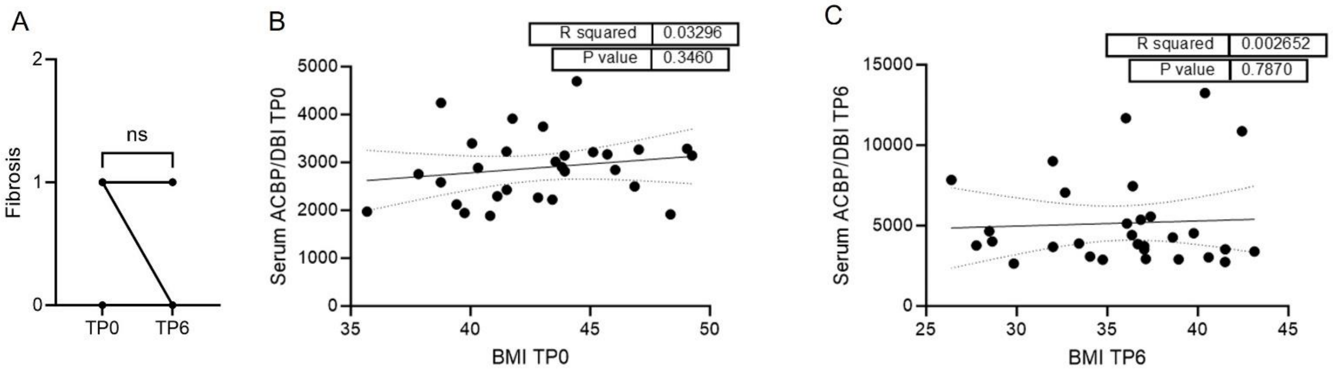

Supplementary Figure S2. (A) Fibrosis score did not differ significantly between baseline and six months post-operatively, n=14; (B&C) BMI and ACBP/DBI are not correlated significantly at TP0 (B) and TP6 (C), n=29 TP0, n=30 TP6; Statistical significance was assessed using a Wilcoxon matched-pairs signed rank test (A) or a simple linear regression to asses if the slope is significantly non-zero (B, C).
